# Hydrogen Sulfide Ameliorates Ischemia/Reperfusion-Induced Hepatitis by Inhibiting Apoptosis and Autophagy Pathways

**DOI:** 10.1155/2014/935251

**Published:** 2014-05-21

**Authors:** Ping Cheng, Fan Wang, Kan Chen, Miao Shen, Weiqi Dai, Ling Xu, Yan Zhang, Chengfen Wang, Jingjing Li, Jing Yang, Rong Zhu, Huawei Zhang, Yuanyuan Zheng, Jie Lu, Yingqun Zhou, Chuanyong Guo

**Affiliations:** ^1^Department of Gastroenterology, The Tenth People's Hospital of Tongji University, Shanghai 200072, China; ^2^Department of Gastroenterology, The Tongren Hospital of Shanghai Jiaotong University, Shanghai 200050, China; ^3^Department of Gastroenterology, Clinical Medical College of the Tenth People's Hospital of Nangjing Medical University, Shanghai 200072, China; ^4^Department of Gastroenterology, The First Affiliated Hospital of Soochow University, Suzhou 215006, China

## Abstract

*Background.* Hepatic ischemia/reperfusion (I/R) injury is an important clinical problem, and its consequences can seriously threaten human health. Apoptosis and autophagy have been shown to contribute to cell death in hepatic I/R injury. Hydrogen sulfide (H_2_S) is the third most common endogenously produced gaseous signaling molecule and is known to exert a protective effect against hepatic I/R injury. In this study, the purpose is to explore both the effect and mechanism of H_2_S on hepatic I/R injury. *Methods.* Balb/c mice were randomized into Sham, I/R, or two doses (14 **μ**mol/kg and 28 **μ**mol/kg) of sodium hydrosulfide (NaHS, an H_2_S donor) preconditioning groups. *Results.* NaHS significantly reduced the levels of TNF-**α** and IL-6 at 12 h and 24 h after injection compared with ischemia/reperfusion challenge alone. The expression of Bcl-2, Bax, Beclin-1, and LC3, which play important roles in the regulation of the apoptosis and autophagy pathways, was also clearly affected by NaHS. Furthermore, NaHS affected the p-JNK1, p-ERK1, and p-p38. *Conclusion.* Our results indicate that H_2_S attenuates hepatic I/R injury, at least in part, by regulating apoptosis through inhibiting JNK1 signaling. The autophagy agonist rapamycin potentiated this hepatoprotective effect by reversing the inhibition of autophagy by H_2_S.

## 1. Introduction

Hepatic ischemia/reperfusion (I/R) injury is an important clinical problem, and usually occurs during liver transplantation, shock, and elective liver resection, and its consequences can seriously threaten human health and daily life [1]. Hepatic I/R injury has been a worldwide health problem in our daily clinical work; thus, the protection of liver against I/R injury has become increasingly important.

There exist complicated mechanisms in the occurrence and development of hepatic I/R injury. At present, more and more evidence show that blocking cell death pathways, such as PI3K/AKT and MAPK [2, 3], can significantly reduce the inflammation caused by hepatic I/R injury [4]. According to the current study, apoptosis, named type I programmed cell death, may be a major cell death of hepatic I/R injury [5]. There are several signal pathways that work in the regulation of apoptosis. Bcl-2 family is considered to act an important role in apoptosis pathway. In the Bcl-2 family, the representative apoptosis-inhibiting genes are Bcl-2 and Bcl-xl, and the proapoptotic genes are Bax and Bad. It has been reported that the balance between Bax and Bcl-2 proteins determines the possibility of cells to survive or to undergo apoptosis after a certain stimulus or injury [6, 7].

In recent years, a new kind of programmed cell death, autophagy, named type II programmed cell death, has attracted attention. Autophagy is first formed in the cytoplasm of the diaphragm (isolation membrane) and wrapped around the damaged cells in the form of autophagy (autophagosome). Autophagosome and lysosomal combine into autophagy-lysosome fusion, which can degrade the contained components. The formation of Autophagosome is a central part of autophagy. It has been confirmed multiple autophagy-related genes involved in the formation of autophagy. Autophagy-related gene protein—Atg6 (Beclin1) can combine with Isolation membrane and raise Atg12-Atg5-Atg16 complex to form pre-autophagic vacuoles. Then Atg8 (LC3-II) binds to Isolation membrane and promotes the extension the outer membrane of Autophagosome, meanwhile Atg12-Atg5-Atg16 complex off to form mature autophagosomes. Autophagy, involving copious aggregations of intracellular autophagosomes, is a cell behavior for surviving harsh environments that has a protective effect [8, 9]. However, when beyond this range, autophagy will finally result in the cell death with the overweening accumulation of autophagosomes, especially under the continuous stimulation of starvation, hypoxia, and inflammation [10]. Our previous study found that hepatic ischemia-reperfusion can activate autophagy and inhibition of autophagy can reduce hepatic I/R injury [4]. But the complex mechanisms involving apoptosis and autophagy underlying the process of hepatic I/R injury are currently a deep and urgent problem; this issue needs further study.

Cystathionine-*β*-synthase (CBS), cystathionine-*γ*-lyase (CSE), and 3-mercapto-pyruvate-sulfur-transferase (MST), which are the primary source of endogenous production of H_2_S, are abundantly expressed in liver [11]. Two-thirds of H_2_S molecules dissociate into hydrogen ions (H+) and bisulfide ions (HS−) under physiological conditions. Therefore, sodium hydrosulfide (NaHS) can be administered as a water-soluble H_2_S donor [12]. H_2_S is known to exert a protective effect against hepatic I/R injury [13]. However, the exact mechanism of this H_2_S effects remains unclear. This study was designed to investigate the role of apoptosis and autophagy in the protective effect of H_2_S against hepatic I/R injury.

## 2. Materials and Methods

### 2.1. Reagents and Drug Preparation

Dimethyl sulfoxide (DMSO), SP600125 (a selective JNK inhibitor), 3-methyladenine (3MA, an autophagy inhibitor), and rapamycin (an autophagy enhancer) and collagenase and DHanks buffer were purchased from Sigma-Aldrich (St. Louis, MO, USA) and stored in the dark at 4°C. 3-(4,5)-Dimethylthiahiazo (-z-y1)-3,5-di-phenytetrazoliumromide (MTT) was purchased from Peptide Institute Inc. (Peptide Institute Inc., Osaka, Japan). An RNA PCR kit was purchased from Takara (Takara Biotechnology, Dalian, China). Dulbecco's modified Eagle's medium and fetal bovine serum were obtained from Invitrogen Corporation (Carlsbad, CA, USA). LC3-II and Beclin-1 were purchased from Abcam, USA. The antibodies (Santa Cruz Biotechnology, CA, USA) used for immunohistochemical staining were antitumor necrosis factor-*α* (TNF-*α*), anti-interleukin 6 (IL-6), anti-JNK, antiphosphorylated JNK (p-JNK), anti-ERK, anti-pERK, anti-p-p38, anti-p38, anti-Bcl-2, and anti-Bax. NaHS was dissolved in saline. SP600125 was solubilized in DMSO at 65 mg/mL. Various final concentrations of NaHS were prepared for different experiments by diluting the stock solution with RPMI-1640.

### 2.2. Animals and Ethics Statement

Male Balb/c mice (6–8 weeks old, 24 ± 2 g) were purchased from Shanghai SLAC Laboratory Animal Co., Ltd. All animals were housed at the Experimental Animal Center, Tongji University, at a constant temperature and with a consistent light cycle (light from 07:00 to 18:00). This study was performed in strict accordance with the recommendations of the Guide for the National Science Council of the Republic of China. The protocol was approved by the Animal Care and Use Committee of the Tenth People's Hospital of Shanghai (permit number: 2011-0111). The study was also approved by the Science and Technology Commission of Shanghai Municipality (ID: SYXK 2007-0006).

### 2.3. Model Establishment and Experimental Design

A model of segmental (70%) hepatic warm ischemia was established using a previously reported method [14, 15].

The mice were allocated randomly to one of four groups, as follows.

Group I, sham group (*n* = 18): mice underwent laparotomy under anesthesia with 1.25% Nembutal (St. Louis, MO, USA), and the abdominal cavity was closed without I/R.

Group II, I/R group (*n* = 18): mice underwent laparotomy under anesthesia with 1.25% Nembutal (St. Louis, MO, USA), and hepatic ischemia was induced by the occlusion of the portal vein, bile duct, and the hepatic artery to the left and median liver lobes with vascular clamps; reperfusion was initiated by the removal of the clamp after 60 min. The mice received an intraperitoneal injection of 1 mL of a physiological solution 30 min before I/R.

Group III, protected group (*n* = 18): mice received an intraperitoneal injection of 1 mL of NaHS solution (14 *μ*mol/kg) 30 min before I/R.

Group IV, protected group (*n* = 18): mice received an intraperitoneal injection of 1 mL of NaHS solution (28 *μ*mol/kg) 30 min before I/R.

The doses were selected on the basis of previous reports and our preliminary experiments. Six mice from each group were randomly selected and were killed 4 h, 12 h, and 24 h after treatment. All serum and liver tissues (median and left lobes) were collected and stored at –80°C for further analysis.

### 2.4. Cell Lines and Culture

Normal human liver cells lines QSG-7701 and LO2 were purchased from the Chinese Academy of Sciences Committee Type Culture Collection cell bank. The two cell lines were cultured in RPMI-1640 culture medium (1640; Thermo, China) supplemented with 10% fetal bovine serum (Hycione, South America), 100 U/mL penicillin, and 100 mg/mL streptomycin (Gibco, Canada) in a humidified incubator at 37°C in 5% CO_2_.

### 2.5. Hepatocyte Isolation

Mouse hepatocytes were isolated by a modified in situ collagenase perfusion technique as described [16]. In brief, the portal vein of male Balb/c mice was cannulated after anesthesia and laparotomy. The liver was perfused with 20 mL prewarmed 37°C DHanks buffer, followed by 20 mL of 0.02% collagenase at a flow rate of 2 mL/min. After perfusion, liver tissues were removed and washed with 20 mL DHanks buffer. The capsule of the liver was removed, and hepatic tissues were dispersed and incubated in 20 mL of 0.01% collagenase in a shaking water bath at 37°C for approximately 30 min. Cell suspension was then filtered through 60-mesh sterile nylon gauze, centrifuged at 500 rpm, and washed three times with RPMI-1640 culture medium. Dispersed hepatocytes were cultured in RPMI-1640 culture medium in a humidified incubator at 37°C and 5% CO_2_. Hepatocyte purity and viability typically exceeded 99% and 95%, respectively.

### 2.6. Cell Proliferation and Viability

Normal human liver cells lines QSG-7701 and LO2 were plated at a density of 2 × 10^4^ cell/well in 96-well plates in 100 *μ*L of medium per well. One day after seeding, the hepatocytes were treated with NaHS (1 *μ*M, 3 *μ*M, 5 *μ*M, 7 *μ*M, or 9 *μ*M) for 30 min. The cells were then treated with hypoxia (3% O_2_, 5% CO_2_, and 92% N_2_) for 24 h and reoxygenation (5% CO_2_, 95% air) for 2 h. Cell viability was measured with an MTT assay and a microplate reader at a wavelength of 490 nm. The experiments were repeated three times.

### 2.7. Biochemical Analysis

After blood collection, the sera were separated by centrifugation at 2000 ×g at room temperature for 10 min. To detect the levels of hepatocellular I/R injury, serum alanine aminotransferase (ALT) and aspartate aminotransferase (AST) were measured with an automated chemical analyzer (Olympus AU1000, Japan).

### 2.8. Measurement of Plasma H_2_S

Aliquots (120 *μ*L) of plasma were mixed with distilled water (100 *μ*L), trichloroacetic acid (10% wt/vol, 120 *μ*L), zinc acetate (1% wt/vol, 60 *μ*L), and N,N-dimethyl-p-phenylenediamine sulfate (20 *μ*mol/L; 40 *μ*L) in 7.2 mol/L HCl and FeCl_3_ (30 *μ*mol/L; 40 *μ*L) in 1.2 mol/L HCl. The absorbance of the resulting solution (670 nm) was measured 10 min thereafter by spectrophotometry (Tecan Systems, San Jose, CA, USA) [17]. H_2_S was calculated against a calibration curve of NaHS (3.125–100 *μ*mol/L). Results showed plasma H_2_S concentrations in the micromolar range.

### 2.9. Assay of Liver H_2_S-Synthesizing Activity

H_2_S-synthesizing activity in liver homogenates was measured as described by Zhang [18]. The H_2_S concentration was calculated against a calibration curve of NaHS. Results were then corrected for the DNA content of the tissue sample [19] and expressed as micromoles of H_2_S formed per microgram of DNA.

### 2.10. Histopathology

When the mice were killed, their liver tissues (median and left lobes) were collected, incubated in 4% paraformaldehyde for at least 24 h, and paraffin blocks were prepared according to the standard protocol. Sections 3 *μ*m thick were cut and stored at room temperature. The paraffin sections were then stained with hematoxylin and eosin (H&E) to observe the level of inflammation and tissue damage with light microscopy.

### 2.11. Immunohistochemistry

The prepared paraffin-embedded sections were dewaxed and rehydrated through a graded series of alcohol and then heated in an oven at 60°C for 20 min. Antigens were recovered by incubating the samples in citrate buffer in a 95°C water bath for 20 min, and then endogenous peroxidase was blocked by incubating them in 3% hydrogen peroxide for 20 min at 37°C. Membranes were ruptured with 0.2% Triton X-100 at room temperature for 30 min and nonspecific binding sites were blocked with 5% bovine serum albumin at 37°C for 20 min, followed by incubation at room temperature for 10 min. The liver slices were then incubated overnight with antibody directed against Beclin-1 (1 : 2000) and LC3–II (1 : 500), p-JNK (1 : 1000), p-ERK (1 : 400), p-p38 (1 : 1000), Bcl-2 (1 : 500), or Bax (1 : 500), and a rabbit anti-mouse antibody at 4°C. Antibody binding was analyzed with a diaminobenzidine kit. The slides were then counterstained with hematoxylin, dehydrated in graded ethanol and xylene, and mounted with Entellan. The slides were observed with light microscopy.

### 2.12. Immunoblotting Analysis

Fresh liver tissues collected from the mice and normal human liver cells lines QSG-7701 and LO2 were rapidly ground in liquid nitrogen and then lysed with RIPA lysis buffer and a protease inhibitor. The protein concentrations were measured with the BCA method. Equivalent amounts of total protein were boiled and subjected to sodium dodecyl sulfate-polyacrylamide gel electrophoresis (SDS-PAGE) using standard methods. Nonspecific binding was blocked with 5% nonfat milk (dissolved in phosphate-buffered saline [PBS]) for 1 h and the blots were then incubated overnight at 4°C with antibodies directed against IL-6 (1 : 500), TNF-*α* (1 : 500), LC3–II (1 : 1000), Beclin-1 (1 : 2000), JNK (1 : 1000), p-JNK (1 : 500), p-ERK (1 : 1000), ERK (1 : 1000), p-p38 (1 : 1000), p-38 (1 : 1000), Bcl-2 (1 : 500), Bax (1 : 500), and *β*-actin (1 : 1000) diluted in 5% milk.

*β*-Actin was used as the internal reference for cytoplasmic proteins. All membranes were washed with PBS + 1% Tween (PBST) and then incubated with a secondary goat anti-mouse or anti-rabbit antibody (1 : 2000) dissolved in PBST, for 45 min at 37°C. Finally, the membranes were washed three times with PBST for 10 min each and the proteins were detected with the Odyssey two-color infrared laser imaging system.

### 2.13. Reverse Transcription-Polymerase Chain Reaction (RT-PCR) and Real-Time PCR

mRNA transcripts were detected and analyzed with quantitative RT-PCR of the liver tissues or cells. Total RNA was extracted from frozen liver tissues with TRIzol reagent (Tiangen Biotech, Beijing, China), as described by the manufacturer. To determine the expression of the target genes, SYBR Green quantitative RT-PCR was performed using a 7900HT fast real-time PCR System (Applied Biosystems, CA, USA), according to the instructions for SYBR Premix Ex Taq (TaKaRa Biotechnology China). The primer sequences are shown in Table 1.

### 2.14. Analysis of Autophagy with Exogenous Green Fluorescent Protein (GFP) LC3 Expression

To monitor the formation of GFP-LC3 puncta, hepatocytes (LO2, QSG7701) were transiently transfected with 1.0 mg of plasmid expressing GFP-LC3 and then treated as follows: normal control (NC), NaHS, anoxia/reoxygenation (A/R), A/R + NaHS, A/R + rapamycin (Rap), NaHS + A/R + Rap, A/R + 3MA, NaHS + A/R + 3MA, or NaHS + A/R + SP600125 (SP). After treatment, the numbers of autophagosomes/cell were counted and are shown in Figure 5(d).

### 2.15. Measurement of Parameters in Nutrient Solution

TNF-*α* and IL-6 were measured with enzyme-linked immunosorbent assay (ELISA) kits for TNF (R&D Systems, MTA00B and M6000B, resp.), according to the manufacturer's instructions.

### 2.16. Transmission Electron Microscopy

Male Balb/c mice were treated as described above, and laparotomy was performed under anesthesia induced with ketamine/xylazine. The liver was flushed with 1 mL of normal sterile saline (NSS) and then perfused with 2 mL of 2.5% glutaraldehyde in PBS. The livers were sectioned and photographed using a transmission electron microscope (Tecnai) at 160 kV Electron Microscopy Film 4489 (Kodak, ESTAR thick base) and printed onto photographic paper. For quantification, 20–30 fields at low magnification (×2500) were randomly selected from each liver, and digital images with scale bars were taken. The numbers of autophagic vacuoles per unit cytoplasmic area (100 *μ*m^2^) were evaluated with the Axio-Vision 4.0 software.

### 2.17. Detection of Apoptosis Using Flow Cytometry

Hepatocytes (LO2, QSG7701) and primary hepatocytes were plated in six-well plates. Cells in the control group, A/R group, and A/R + NaHS-treated group were collected after 24 h, washed twice in cold PBS, mixed with 100 *μ*L of 1 × binding buffer, and incubated with the Annexin V Apoptosis Detection Kit (containing annexin V-fluorescein isothiocyanate [FITC], propidium iodide [PI] solution, and annexin-V-binding buffer) at room temperature for 15 min. Cell apoptosis was assessed with FITC (BD Pharmingen, San Jose, CA, USA). A flow-cytometric analysis was performed on cells that were in the early apoptosis (annexin V^+^/PI^−^) or late apoptosis/necrosis (annexin V^+^/PI^+^) phase.

### 2.18. Terminal Deoxynucleotidyl Transferase dUTP Nick End Labeling (TUNEL) Staining

The sections were deparaffinized and rehydrated; TUNEL staining was then performed according to the instructions for the TUNEL assay kit (Roche, 11684795910). The sections were counterstained with hematoxylin and the total hepatocytes and TUNEL-positive cells were quantified with light microscopy.

### 2.19. Statistical Analysis

All results are expressed as means ± SD. Comparisons between two groups were made with Student's *t*-test. Statistical differences in multiple groups were determined by multiple comparisons with analysis of variance, followed by Tukey's post hoc test. *P* < 0.05 was considered statistically significant. All statistical analyses were performed with SPSS 17.0 for Windows.

## 3. Results

### 3.1. H_2_S Pretreatment Ameliorates Hepatic I/R Injury

**Figure 1:**
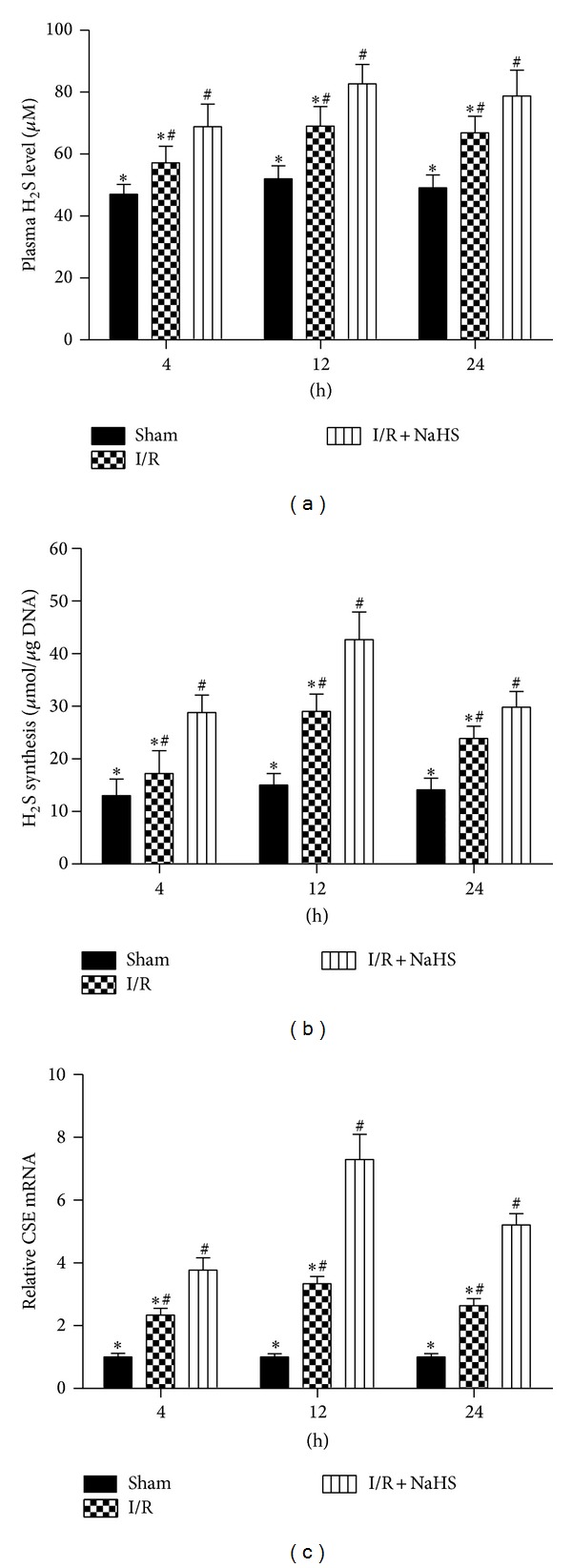
Plasma H_2_S level, H_2_S-synthesizing activity, and CSE mRNA expression in livers. (a) Plasma hydrogen sulfide (H_2_S) concentration was expressed as the mean ± SD of 6 animals per group. *P < 0.05 for saline versus saline + I/R, ^#^ *P* < 0.05 for saline + I/R versus I/R + NaHS (14 *μ*mol/kg). (b) Liver H_2_S-synthesizing activity was expressed as the mean ± SD of 6 animals per group. *P < 0.05 for saline versus saline + I/R, ^#^ *P* < 0.05 for saline + I/R versus I/R + NaHS (14 *μ*mol/kg). (c) The mRNA expression of CSE was detected by real time PCR. *P < 0.05 for saline versus saline + I/R, ^#^ *P* < 0.05 for saline + I/R versus I/R + NaHS (14 *μ*m/kg).

**Figure 2:**
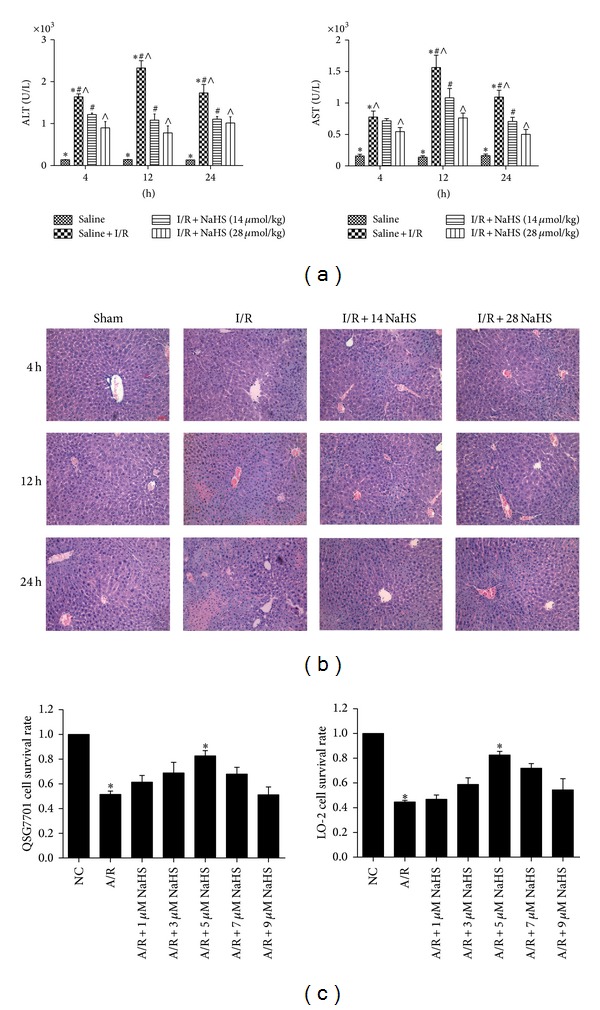
Effect of NaHS on hepatic ischemia-reperfusion injury. (a) The serum ALT and AST levels were expressed as the mean ± SD of 6 animals per group. *P < 0.05 for saline versus saline + I/R, ^#^ *P* < 0.05 for saline + I/R versus I/R + NaHS (14 *μ*mol/kg), and ^∧^ *P* < 0.05 for saline + I/R versus I/R + NaHS (28 *μ*mol/kg). (b) Representative hematoxylin and eosin (H&E) stained sections of liver. Original magnifications: ×200. (c) Hepatocytes (LO2, QSG7701) that were subjected to NaHS (1 *μ*M, 3 *μ*M, 5 *μ*M, 7 *μ*M, and 9 *μ*M), then the cells were treated with 24 h hypoxia (3% O_2_, 5% CO_2_, and 92% N_2_) and 2 h reoxygenation (5% CO_2_, 95% air). Cell viability was measured using an MTT assay and a microplate reader at a wavelength of 490 nm. The experiments were repeated three times. The results are expressed as the mean ± SD (*n* = 5). *P < 0.05 for (A/R + 5 *μ*M NaHS) versus saline + A/R.

**Figure 3:**
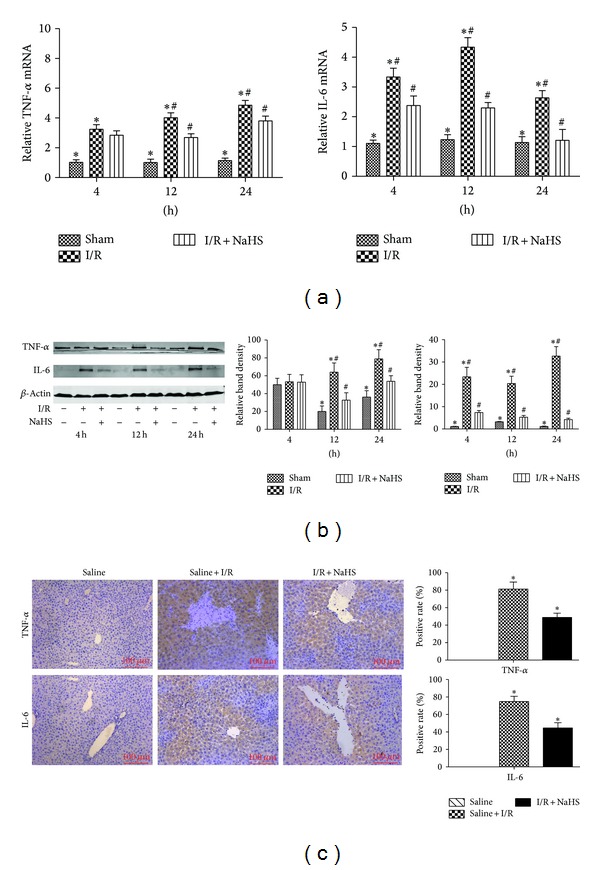
NaHS pretreatment inhibits the release of cytokines during hepatic ischemia-reperfusion (I/R) injury. (a) The mRNA expression of IL-6 and TNF-*α* in liver tissues was detected by real time PCR. *P < 0.05 for saline versus saline + I/R, ^#^ *P* < 0.05 for saline + I/R versus I/R + NaHS (14 *μ*mol/kg). (b) Western blots and quantitative evaluation of the expression of IL-6 and TNF-*α* in liver tissues with *β*-actin as protein loading control. *P < 0.05 for saline versus saline + I/R, ^#^ *P* < 0.05 for saline + I/R versus I/R + NaHS (14 *μ*mol/kg). (c) Immunohistochemistry staining (200x) showed the expression of TNF-*α* and IL-6 in liver tissue at 12 h. *P < 0.05 for (saline + I/R) versus (I/R + NaHS).

We performed an assay using a hepatic I/R injury model. Mice were injected intraperitoneally with either NSS or NaHS (14 *μ*mol/kg or 28 *μ*mol/kg). Hepatic I/R injury resulted in a significant increase in the plasma H_2_S level compared with the sham group and NaHS preconditioning significantly increase in the plasma H_2_S level compared with the I/R group (*P < 0.05, Figure 1(a)). The amounts of H_2_S formed in liver after I/R were significantly enhanced compared with the sham group, and NaHS preconditioning further increased it compared with the I/R group (*P* < 0.05, Figure 1(b)). In addition, the expression level of CSE mRNA was significantly increased in the liver of mice subjected to I/R compared with that in the sham group (*P* < 0.05, Figure 1(c)). Liver function was assessed after NaHS treatment by measuring the serum levels of ALT and AST. As shown in Figure 2(a), the levels of ALT and AST clearly increased after I/R in the model group compared with that in the saline-treated group, and NaHS pretreatment significantly attenuated the I/R-induced increases in serum ALT and AST at 4 h, 12 h, and 24 h after injury (*P* < 0.05). We also examined the histopathological changes in the liver tissues. The pathological features of the liver tissues from the three groups after H&E staining are shown in Figure 2(b). The structures of the liver tissues were completely maintained and remained ordered in the saline group, whereas a disordered lobular structure, marked hepatocyte necrosis, and polymorphonuclear cell infiltration were observed in the I/R-treated model group at 8 h, 12 h, and 24 h after I/R injury. Pretreatment with NaHS (14 *μ*mol/kg) clearly attenuated these pathological changes at 12 h. Therefore, we administered NaHS at a concentration of 14 *μ*mol/kg in vivo in subsequent experiments. I/R-induced hepatic injury is associated with changes in the levels of inflammatory cytokines. We demonstrated with real-time PCR and immunoblotting that the expression of TNF-*α* and IL-6 was significantly elevated in the model group relative to their expression in the saline group (*P* < 0.05; Figures 3(a) and 3(b)). We also investigated the locations and levels of TNF-*α* and IL-6 with immunohistochemical staining. Both were expressed markedly more strongly in the model group than in the saline group at 12 h. In contrast, after NaHS treatment, there was a significant reduction in the specific areas in which they were expressed (*P < 0.05; Figure 3(c)).

To confirm the hepatoprotective effect of H_2_S, hepatocytes were pretreated with increasing concentrations of NaHS in cell proliferation and cytotoxicity (MTT) assays to assess their viability in vitro. As shown in Figure 2(c), NaHS (5 *μ*M) preconditioning significantly ameliorated hepatic I/R injury (*P < 0.05). Based on these experimental data, we used 5 *μ*M NaHS in vitro for subsequent experiments.

### 3.2. H_2_S Attenuates Hepatocyte Apoptosis In Vitro and In Vivo

**Figure 4:**
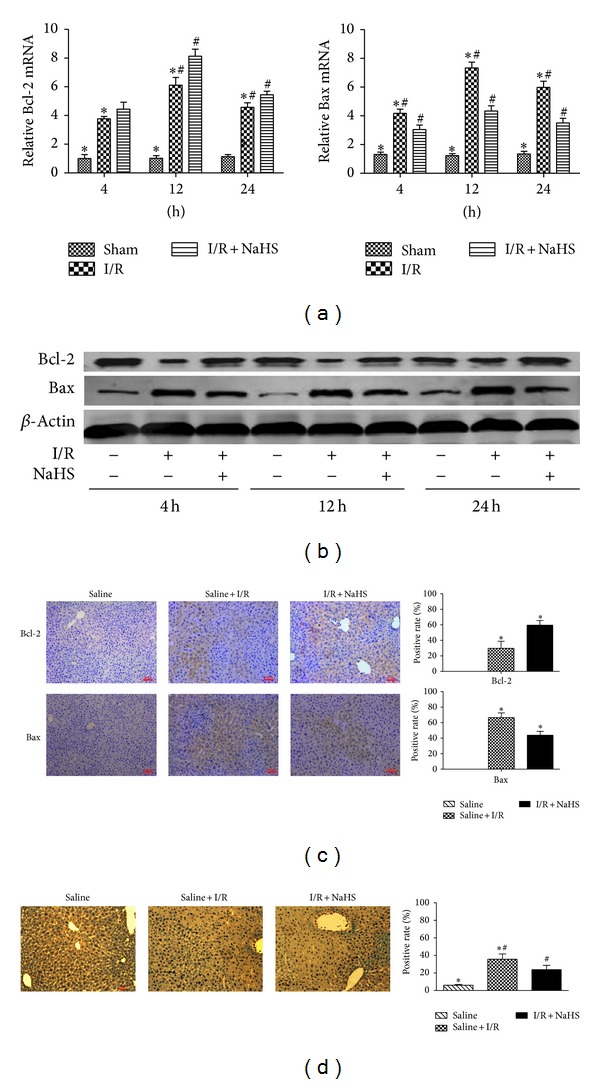
Effect of NaHS pretreatment on regulation of apoptosis in vivo. (a) The mRNA expression of Bcl-2 and Bax in liver tissues was detected by real time PCR. *P < 0.05 for saline versus saline + I/R, ^#^ *P* < 0.05 for saline + I/R versus I/R + NaHS (14 *μ*mol/kg). (b) Western blots and quantitative evaluation of the expression of Bcl-2 and Bax in liver tissues. *P < 0.05 for saline versus saline + I/R,^#^ *P* < 0.05 for saline + I/R versus I/R + NaHS (14 *μ*mol/kg). (c) Immunohistochemistry staining (200x) showed the expression of Bcl-2 and Bax in liver tissue at 12 h. *P < 0.05 for saline + I/R versus I/R + NaHS. (d) TUNNEL staining showed the apoptotic cells in three groups at 12 h. Original magnifications: ×200. *P < 0.05 for saline versus saline + I/R, ^#^ *P* < 0.05 for saline + I/R versus I/R + NaHS.

As previously noted, apoptotic cell death occurs after hepatic I/R injury, causing hepatic dysfunction. Therefore, we next investigated the effects of H_2_S on the inhibition of apoptosis using real-time PCR, immunoblotting analysis, and immunohistochemical staining during hepatic I/R injury. As expected, NaHS pretreatment attenuated hepatocyte apoptosis by increasing Bcl-2 and reducing Bax levels compared with those in the I/R group (*P < 0.05; Figure 4). TUNEL staining was used to identify the effects of NaHS on hepatocyte apoptosis. As showed in Figure 4(d), NaHS preconditioning (14 *μ*mol/kg) markedly reduced the TUNEL index (*P < 0.05). There was a similar attenuation of hepatocyte apoptosis in the A/R hepatocytes analyzed with immunoblotting and flow cytometry (*P < 0.05; Figures 6(b) and 6(c)). Reductions in the percentages of cells in early apoptosis (quadrant 2) and late apoptosis (quadrant 3) were identified in the hepatocytes after treatment with NaHS (5 *μ*M).

### 3.3. H_2_S Attenuates Autophagy In Vitro and In Vivo

**Figure 5:**
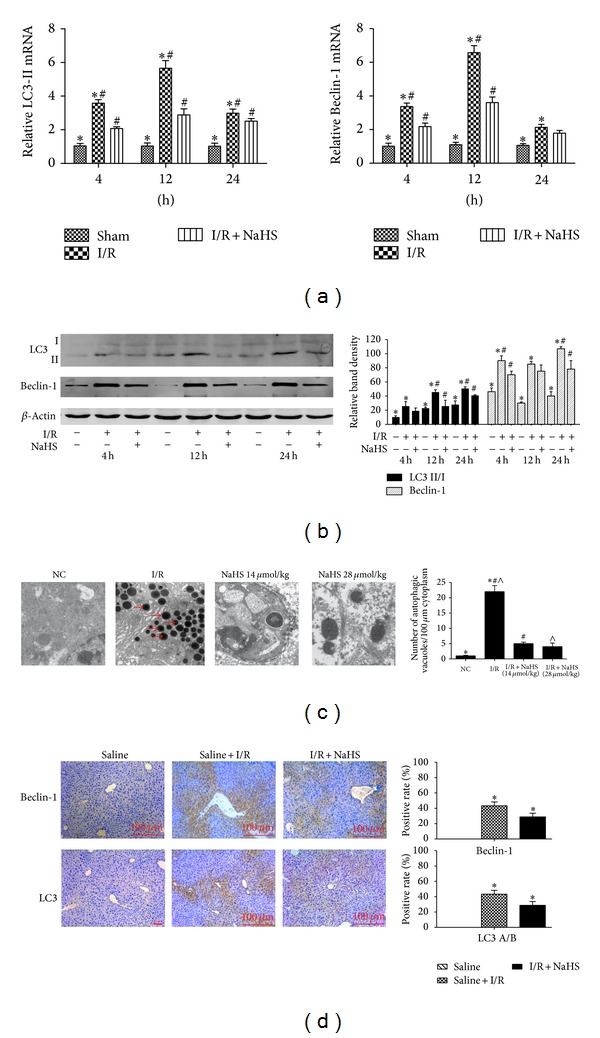
Effect of NaHS pretreatment on regulation of autophagy in vivo. (a) The mRNA expression of Beclin-1 and LC3 in liver tissues was detected by real time PCR. *P < 0.05 for saline versus saline + I/R, ^#^ *P* < 0.05 for saline + I/R versus I/R + NaHS (14 *μ*mol/kg). (b) Western blots and quantitative evaluation of the expression of Beclin-1 and LC3 in liver tissues with *β*-actin as protein loading control. *P < 0.05 for saline versus saline + I/R, ^#^ *P* < 0.05 for saline + I/R versus I/R + NaHS (14 um/kg). (c) Electron microscopy showed the ultrastructure and autophagosomes (“→” indicated the autophagosomes). Original magnifications: ×2500. *P < 0.05 for saline versus saline + I/R, ^#, ∧^ *P* < 0.05 for saline + I/R versus I/R + NaHS. (d) Immunohistochemistry staining (200x) showed the expression of Beclin-1 and LC3 protein in liver tissue at 12 h. *P < 0.05 for saline + I/R versus I/R + NaHS.

It is well known that I/R-induced hepatitis can involve autophagy. LC3 is an important marker of autophagy, and Beclin-1 plays an important role in the regulation of autophagy. To further assess the activation of autophagy by NaHS pretreatment in our models of I/R and A/R, the expression of LC3 and Beclin-1 in liver tissues and hepatocytes was determined with real-time PCR and immunoblotting (Figures 5(a), 5(b), and 7(a)). These results indicate that the levels of LC3 and Beclin-1 were significantly reduced after NaHS preconditioning during I/R-induced hepatitis. The formation of autophagosomes is a pivotal process in autophagy, so we used electron microscopy to observe the ultrastructures of the treated hepatic cells (Figure 5(c)). Autophagic vacuoles were dramatically increased in the model group compared with the basal levels in the sham control group. However, after NaHS treatment, the liver nuclear chromatin was more homogeneous, and the integrity of the cell structure was still intact. Analysis of the immunohistochemical changes in the mouse livers confirmed these results (*P < 0.05; Figure 5(d)). We also detected the formation of fluorescent puncta or autophagosomes in hepatocytes expressing exogenous GFP-LC3. The control hepatocytes showed occasional GFP-LC3-stained puncta (Figure 7(b)), and there were almost no GFP-LC3-stained puncta in the NaHS-treated hepatocytes. In the hepatocytes pretreated with A/R, some cells displayed numerous unevenly distributed cup- or ring-shaped green dots of various sizes, whereas NaHS markedly reduced the numbers of autophagosomes in the cells. These results indicate that A/R increased the number of GFP-LC3-positive autophagosomes from the basal level, and this was reversed by NaHS pretreatment.

### 3.4. H_2_S Attenuates JNK Signaling after Hepatic I/R

Because JNK signaling plays a key role in regulating the inflammatory responses [20], to further I/R-induced hepatitis using immunoblotting and immunohistochemistry, I/R and A/R upregulated the levels of p-JNK, and NaHS preconditioning downregulated JNK1 and ERK phosphorylation compared with that in the I/R and A/R groups, whereas p-ERK remained at baseline levels in the nonpretreated animals (control and I/R injury) (Figures 8(a) and 8(c)). The phosphorylation of p38 was similar in all the groups, with no significant differences. We also investigated the phosphorylation levels of JNK1 in vitro (*P < 0.05; Figure 8(b)). Both NaHS and A/R upregulated phosphorylation levels of JNK1 in hepatocytes compared with its expression in the sham control. The administration of NaHS + A/R reduced this increase in p-JNK1. These results indicate that NaHS interrupts A/R-induced hepatitis by attenuating the JNK signaling pathways.

### 3.5. JNK1 Inhibition Enhances the Hepatoprotective Effect of H_2_S

**Figure 6:**
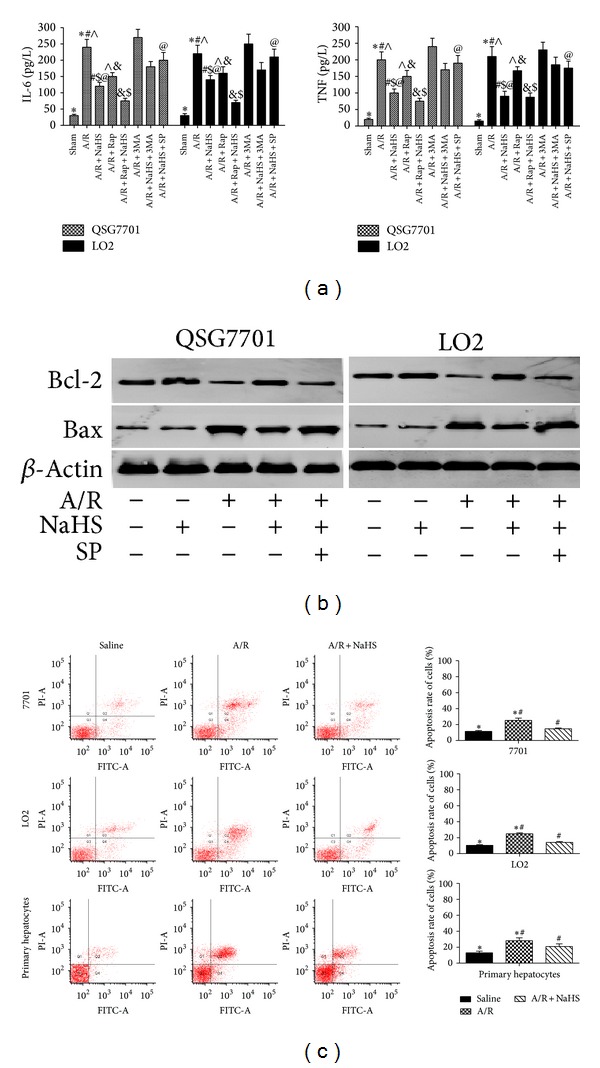
Effect of NaHS pretreatment on regulation of apoptosis in vitro. (a) IL-6 and TNF levels were assessed by ELISA. Data are expressed as mean ± SD of 5 wells nutrient solution per group. *Significant difference from control, *P* < 0.05; ^#^significant difference from A/R group, *P* < 0.05; ^∧^ *P* < 0.05 for A/R versus A/R + Rap; ^$^ *P* < 0.05 for A/R + NaHS versus A/R + NaHS + Rap; ^@^ *P* < 0.05 for A/R + NaHS versus A/R + NaHS + SP. (b) Western blots of the expression of Bcl-2 and Bax in vitro that were subjected to saline, A/R, A/R + NaHS, and A/R + NaHS + SP with *β*-actin as protein loading control. (c) Flow cytometric analyses of annexin-V/PI staining of hepatocytes (LO2, QSG7701, and primary hepatocytes). *P < 0.05 for saline versus saline + A/R, ^#^ *P* < 0.05 for saline + A/R versus A/R + NaHS.

To assess the relevance of the activation of the JNK pathway to the protective effects of H_2_S, hepatocytes (LO2, QSG7701) were treated with the JNK1 inhibitor SP600125 (10 *μ*M) [21]. The administration of SP600125 significantly reduced the increase in JNK1 phosphorylation (*P < 0.05; Figure 8(b)) and enhanced the hepatoprotective effects of H_2_S (*P < 0.05; Figure 6(a)).

## 4. Discussion

Hepatic I/R injury, as a common clinical problem, has already attracted the attention of scientists worldwide. Recently, several studies have reported that H_2_S displays anti-inflammatory and cytoprotective activity in ischemic and hypoxic injury [22]. However, the underlying mechanism remains largely unknown. According to the current study, apoptosis may be one of major ways of cell death of hepatic I/R injury [5]. It is well known that Bax promotes apoptosis, and conversely, Bcl-2 inhibits apoptosis by blocking the release and oligomerization of Bax. The balance between the Bax and Bcl-2 proteins has also been linked to the induction of apoptotic cell death after I/R [6, 23]. In our study, the results showed that hepatic I/R increase of Bax and decrease of Bcl-2 finally resulted in the cell death. However, H_2_S preconditioning makes the balance between Bax and Bcl-2 trended to normal, with the upregulation of Bcl-2 and downregulation of Bax (Figures 4(a), 4(b), and 4(c)). Meanwhile, the number of TUNEL-positive hepatic cells had a significant decrease (Figure 4(d)). Hence, we supposed that H_2_S ameliorated cell death in hepatic I/R injury by inhibiting apoptosis.

JNK1 is one of the most potent cell-survival signaling pathways and is known to play an essential role in hepatic injury. Consequently, the modulation of this pathway may offer a potential strategy to reduce organ damage during liver injury in the clinical context [24]. Tsung et al. have reported that I/R liver injury can activate JNK signaling [25] and other studies have demonstrated that inhibition of JNK activation attenuates I/R-induced hepatocyte apoptosis [24]. However, whether the attenuation of JNK activity is the key mechanism in the protection conferred by H_2_S preconditioning against liver I/R injury remained unclear. In our study, the results showed that H_2_S preconditioning attenuated JNK signaling after hepatic I/R (Figure 8) and the hepatoprotective effect of H_2_S against hepatic I/R injury were enhanced by JNK1 inhibitor SP600125 (Figures 6(a) and 6(b)). Therefore, we inferred that the hepatoprotective effect of H_2_S against hepatic I/R injury depends, at least in part, upon the inhibition of apoptosis by JNK pathway.

**Figure 7:**
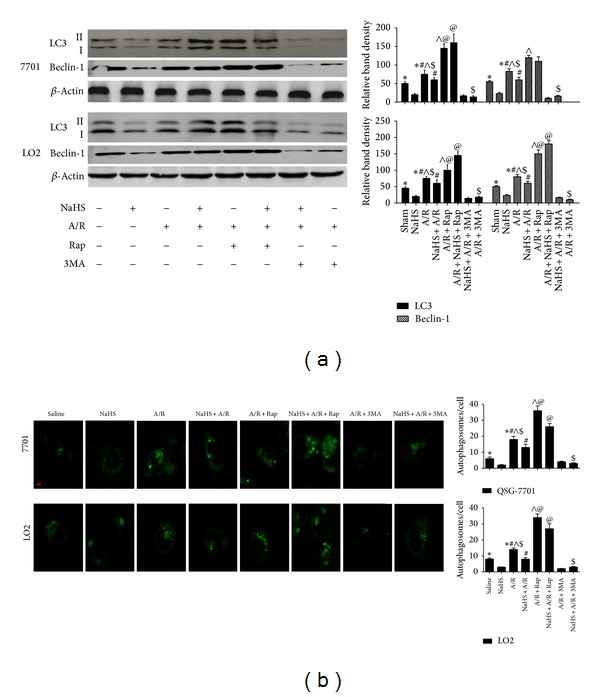
Effect of NaHS pretreatment on regulation of autophagy in vitro. (a) Western blots and quantitative evaluation of the expression of Beclin-1 and LC3 in vitro with *β*-actin as protein loading control. *Significant difference from control, *P* < 0.05; ^#^significant difference from A/R group, *P* < 0.05; ^∧^ *P* < 0.05 for A/R versus A/R + Rap; ^$^ *P* < 0.05 for A/R + NaHS versus A/R + NaHS + Rap; ^@^ *P* < 0.05 for A/R + NaHS versus A/R + NaHS + SP. (b) The average number of autophagosomes/cell ± SD counted from confocal microscopy images of hepatocytes (LO2, QSG7701) expressing GFP-LC3. (Original magnifications: ×400.)

Evankovich et al. have reported that I/R-induced hepatitis is mediated by autophagy [26]. A certain degree of autophagy is an essential cellular self-help behavior in harsh environments [8, 27] and beyond this range will lead to cell damage. Our previous study results showed hepatic I/R can activate autophagy, and inhibition of autophagy can reduce hepatic I/R injury [4]. In this study, we found that LC3-II and Beclin-1 are inhibited by H_2_S preconditioning during I/R-induced hepatitis in vitro and in vivo (Figures 5 and 7). As LC3-II is an important autophagy landmark and upregulation of Beclin-1 promotes the process of autophagy [28], H_2_S attenuates hepatic I/R injury by downregulating the process of autophagy. And the reduction of autophagosomes in H_2_S treatment group under the electron microscope further confirmed our thinking (Figure 5(c)). However, there are mutual influences between apoptosis and autophagy; apoptosis is inhibited when autophagy is activated, whereas inhibition of autophagy can promote cell death and the activity of caspase proteins [29]. Recent evidence supports the view that enhancing autophagy may be a new method to reduce hepatic I/R injury, and such a hepatoprotective role of autophagy may be related to its antiapoptotic and anti-inflammatory activity [30]. For example, Bcl-2 has been identified as a direct binding partner of Beclin-1, reducing the cell's autophagic activity [31]. In resting cells, Bcl-2 is constitutively bound to Beclin-1, thus allowing only low (basal) levels of autophagy. Under autophagy-inducing conditions, Bcl-2 dissociates from Beclin-1, resulting in increased autophagy [31]. We wondered whether the activation of autophagy enhances the hepatoprotective effect of H_2_S. Further experiments confirmed that further reduction of autophagy with 3MA (an autophagy inhibitor) diminished the protective effect of H_2_S, whereas rapamycin (an autophagy enhancer) reversed the autophagy-inhibitory effect and consequently enhanced the protective effect of H_2_S against I/R- and A/R-induced hepatic injury (Figures 6(a) and 7). Interestingly, it seems to be a contradictory result. Autophagy, an important protective mechanism against I/R- and A/R-induced hepatic injury, was inhibited by H_2_S, and H_2_S played a protective role against I/R- and A/R-induced hepatic injury. However, reversing the autophagy inhibitory effect of H_2_S with rapamycin could be used to potentiate this hepatoprotective effect. This indicates that the protective mechanism of H_2_S is multifaceted.

**Figure 8:**
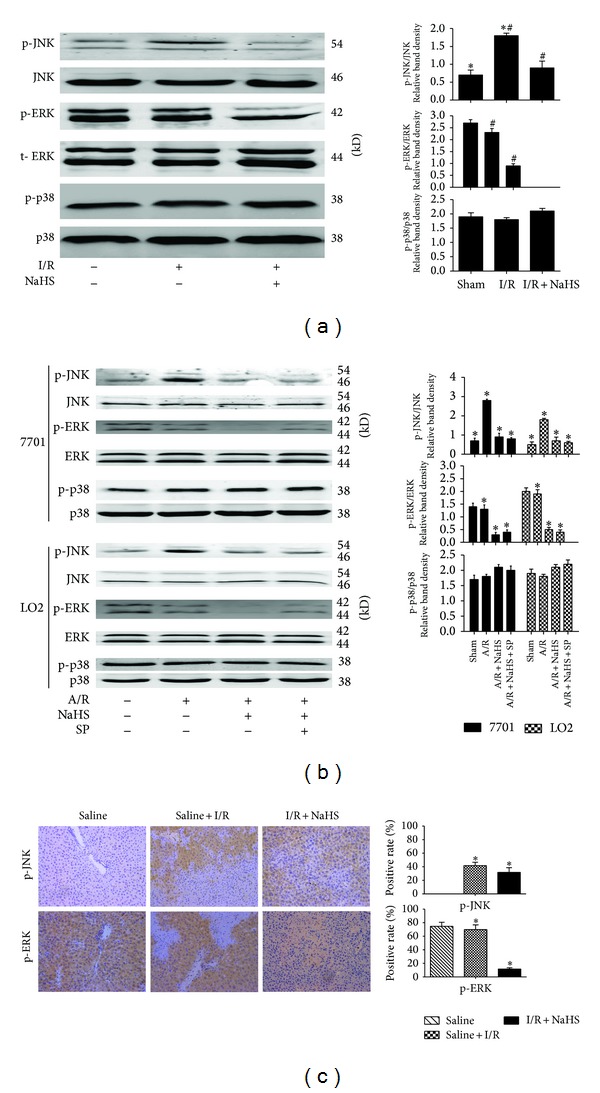
The effect of JNK1 in the protective effect of H_2_S on hepatocyte A/R and I/R injuries. (a) Western blots and quantitative evaluation of the expression of p-JNK, p-ERK, and p-p38 in vivo. *P < 0.05 for saline versus saline + I/R, ^#^ *P* < 0.05 for saline + I/R versus I/R + NaHS. (b) Western blots and quantitative evaluation of the expression of p-JNK, p-ERK, and p-p38 in vitro. *P < 0.05. (c) Immunohistochemistry staining (200x) showed the expression of p-JNK and p-ERK in liver tissue at 12 h. *P < 0.05 for saline + I/R versus I/R + NaHS.

**Figure 9:**
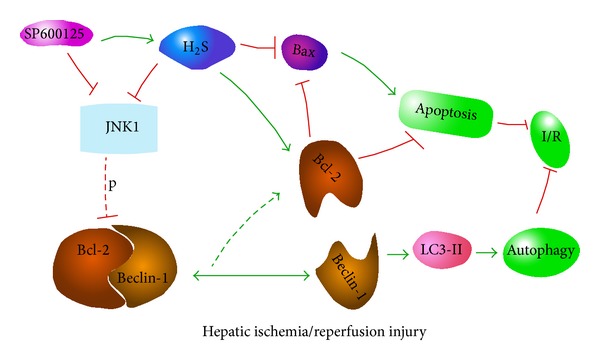
In the condition of hepatic ischemia-reperfusion injury, H_2_S reduces autophagy (which is an important protective mechanism against I/R- and A/R-induced hepatitis) through the suppression of the JNK pathway. However, it also plays an antiapoptotic role in ameliorating I/R- and A/R-induced hepatitis and these protective effects are enhanced by the inhibition of JNK. Our findings show that, although autophagy was inhibited by H_2_S, H_2_S still showed a protective effect against I/R. Furthermore, the JNK1-mediated phosphorylation of Bcl-2 substantially reduced the affinity of Bcl-2 for Beclin-1, leading to its rapid dissociation from Beclin-1 and the subsequent induction of prosurvival autophagy, reducing hepatic I/R injury. In our study, the reduced phosphorylation of JNK1 (compared with that in the I/R and A/R groups) by preconditioning with NaHS may have weakened the process described above.

As noted above, the hepatoprotective effect of H_2_S against hepatic I/R injury depends, at least in part, on weakening the apoptosis by JNK pathways. However, autophagy is also inhibited by H_2_S. A recent paper reported that the JNK1 inhibitor SP600125 blocks autophagy, whereas ERK1 and p38 inhibitors have no effect on it [32]. In our study, an immunoblotting analysis showed that I/R and A/R upregulated the levels of p-JNK1 (not p-ERK1) and that preconditioning with NaHS deregulated JNK1 and ERK1 phosphorylation compared with that in the I/R and A/R groups. The phosphorylation of p38 was similar in all groups, with no significant differences (Figure 8). In turn, the JNK inhibitor SP600125 enhanced the hepatoprotective effects of H_2_S (Figure 6(a)). Therefore, the JNK pathway may work as a double-edged sword in H_2_S preconditioning during I/R- and A/R-induced liver injury. H_2_S reduces autophagy (which is an important protective mechanism against I/R- and A/R-induced hepatitis) through the suppression of the JNK pathway. However, it also plays an antiapoptotic role in ameliorating I/R- and A/R-induced hepatitis and these protective effects are enhanced by the inhibition of JNK. This may be used to interpret our findings that although autophagy was inhibited by H_2_S, H_2_S still showed a protective effect against I/R. Furthermore, the JNK1-mediated phosphorylation of Bcl-2 substantially reduced the affinity of Bcl-2 for Beclin-1, leading to its rapid dissociation from Beclin-1 and the subsequent induction of prosurvival autophagy, reducing hepatic I/R injury [33]. In our study, the reduced phosphorylation of JNK1 (compared with that in the I/R and A/R groups) by preconditioning with NaHS may have weakened the process described in Figure 9.

## 5. Conclusion

In summary, our findings demonstrate that H_2_S protects against I/R- and A/R-induced liver injury, at least in part, by weakening the apoptosis through the suppression of JNK Signaling pathway. The autophagy agonist rapamycin can be used to potentiate this hepatoprotective effect by reversing the inhibition of autophagy by H_2_S. Therefore, enhancing autophagy may be a promising strategy to improve the hepatoprotective capacity of H_2_S against I/R- and A/R-induced liver injury. Currently, research into the molecular mechanisms of hepatic injury and repair and intervention strategies using animal experiments has made some progress. Clinical evidence-based research should also be useful in further exploring the little-understood field of liver injury.

## Figures and Tables

**Table 1:** 

Gene		Primer sequence (5′→3′)
Beclin-1	Forward	AGATGCCTCCCCGATCAGAG
	Reverse	CTTACCACAGCCCAGGCGAA

TNF-*α*	Forward	CAGGCGGTGCCTATGTCTC
	Reverse	CGATCACCCCGAAGTTCAGTAG

*β*-actin	Forward	GGCTGTATTCCCCTCCATCG
	Reverse	CCAGTTGGTAACAATGCCATGT

Bcl-2	Forward	GCTACCGTCGTGACTTCGC
	Reverse	CCCCACCGAACTCAAAGAAGG

LC3	Forward	TGCTGTCCCGAATGTCTCCTG
	Reverse	GCTAACCAAGCCTTCTTCCTCC

IL-6	Forward	CTGCAAGAGACTTCCATCCAG
	Reverse	AGTGGTATAGACAGGTCTGTTGG

Bax	Forward	AGACAGGGGCCTTTTTGCTAC
	Reverse	AATTCGCCGGAGACACTCG

CSE	Forward	GCAGCGATTACACCACAAACC
	Reverse	AATATCAGCACCCAGAGCCAAAG
